# Revolutions in Chemistry: Assessment of Six 20th Century Candidates (The Instrumental Revolution; Hückel Molecular Orbital Theory; Hückel’s 4*n* + 2 Rule; the Woodward–Hoffmann Rules; Quantum Chemistry; and Retrosynthetic Analysis)

**DOI:** 10.1021/jacsau.3c00278

**Published:** 2023-08-28

**Authors:** Jeffrey I. Seeman

**Affiliations:** Department of Chemistry University of Richmond, Richmond, Virginia 23173, United States

**Keywords:** revolutions in chemistry, history of chemistry, instrumental revolution, theory and computational chemistry, taxonomic and lexiconic changes

## Abstract

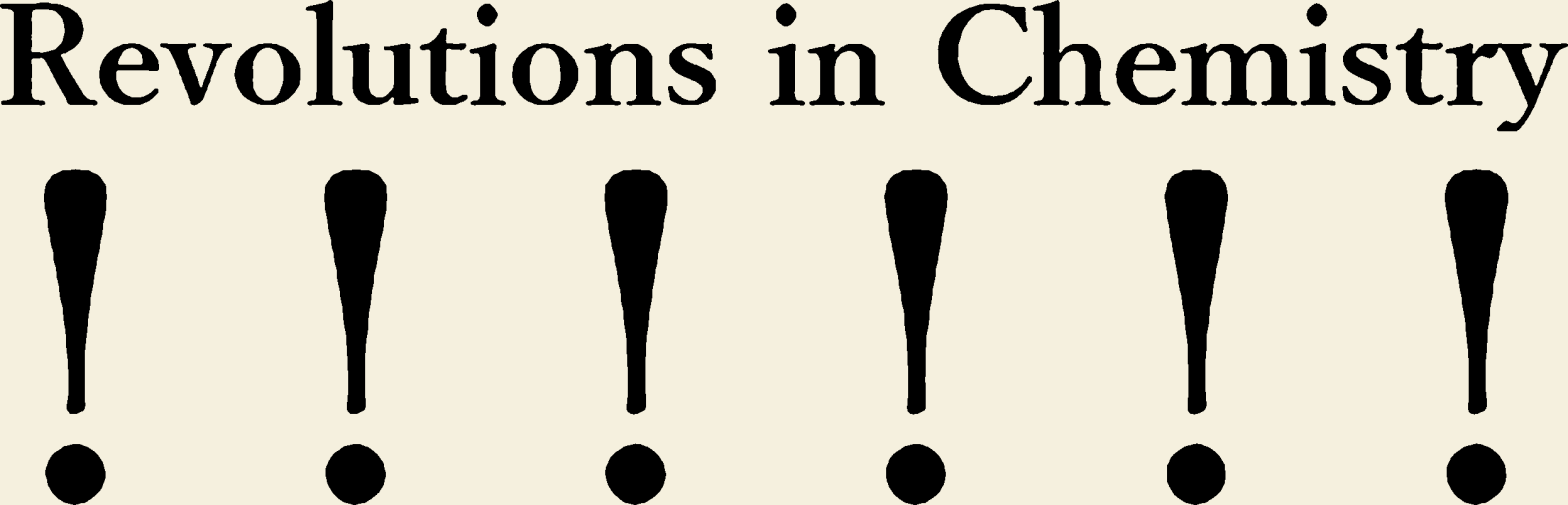

Six 20th century candidates for revolutions in chemistry are examined, using a definitional scheme published recently by the author. Six groupings of 13 characteristics of revolutions in science are considered: causes and birthings of revolutions, relationships between the old and the new, conceptual qualities of the candidate revolutions, instrumental and methodological functions, social construction of knowledge and practical considerations, and testimonials. The Instrumental Revolution was judged to be a revolution in chemistry because of the enormous increase in community-wide knowledge provided by the new instruments and the intentionality in the identification of specific target instruments, in the mindfulness in their design, manufacture, testing, use, and ultimately commercialization. The Woodward–Hoffmann rules were judged to precipitate the Quantum Chemistry Revolution because of theoretical, practical, and social construction of knowledge characteristics. Neither Hückel molecular orbital theory nor Hückel’s 4*n* + 2 rule was considered an initiator of a revolution in chemistry but rather participants in the Quantum Chemistry Revolution. Retrosynthetic analysis was not judged to initiate a revolution in chemistry.

## 1 Preface

Revolutions in science are a well-known feature in the history and philosophy of science.^1−21^ Think Lavoisier, phlogiston, and the First Revolution in Chemistry. Think the Industrial Revolution. Indeed, studies of the progress of science, its causes, and its manifestations help scientists place their own achievements and those of their colleagues and heroes within a larger context. Such deep thinking may help aspiring young scientists or even midcareer scientists make more informed decisions about their own educational and career trajectories. Studies that distinguish between revolutionary and merely important, even seminal achievements in chemistry can be revealing and centering. In doing so, scientists can see the history of their own science within a rational framework.

## 2 Introduction

Despite the voluminous discussion of revolutions in chemistry over many decades by many researchers, there is no uniformly agreed upon definition of the phenomenon nor any process to compare and analyze a set of candidate revolutions.^22^ Historians and philosophers of chemistry have generally described certain handpicked episodes in the history of chemistry as being (and far less frequently, not being) revolutions in chemistry. Typically, these scholars have used characterizations that are idiosyncratic to each specific episode.^23−26^

Recently I developed a method based on the enormous literature on revolutions in science by which one can examine several candidates as revolutions in chemistry in a comparative fashion.^22^ This method is quite flexible, allowing scholars to examine past and current candidates and to peek a bit forward in time, i.e., the method has predictive capabilities and is portable.^27,28^ This publication is Part III of a three-part series on revolutions in chemistry, in particular, and revolutions in science, in general.

- Part 1: I published *Revolutions in Science, Revolutions in Chemistry* in 2023 in the philosophy of chemistry journal *Foundation of Chemistry*.^22^
- Part 2: I published *Understanding Chemistry: from ‘Heuristic (Soft) Explanations and Reasoning by Analogy*’ *to ‘Quantum Chemistry* with Dean Tantillo^29^ in 2022 in *Chemical Science*.
- Part 3: This article, *Revolutions in Chemistry: Assessment of Six 20th Century Candidates (The Instrumental Revolution; Hückel Molecular Orbital Theory; Hückel’s 4n+2 Rule; the Woodward–Hoffmann Rules; Quantum Chemistry; and Retrosynthetic Analysis)*.

In Part 1, I proposed the first definition of revolutions in chemistry based on the characteristics reported and used in more than one hundred publications and books on the subject.^22^ This involved identifying characteristics of revolutions of science reported in this broad and 60-year-old literature. After removing duplicates and merging similar characteristics, I identified 13 unique characteristics of revolutions in science and, after further detailed analysis, assembled these 13 characteristics into six independent factors (Table 1). A more complete description of the characteristics of a revolution in science, i.e., a more complete Table 1, appears in Part 1 of this series.^22^Table 1 should be considered to be a working document, to which modifications can and should be made based on experience and context.

Recently, Dean Tantillo and I concluded that there was a conceptual discontinuity in the progress of chemistry, from heuristic explanations and reasoning by analogy to explanations based on quantum chemistry. That collaboration led to Part 2 in this trilogy.^29^

In this publication (Part 3 of the series), the conceptual framework from Part 1, namely the newly derived definition of revolutions in science and in chemistry, is used to analyze and judge six candidate revolutions in chemistry. This is the first publication on revolutions in chemistry in which the characteristics of this phenomenon are clearly defined and in which several, in this instance, six, candidates are evaluated and compared.

The choice of candidates for this study was based on the following considerations. I wanted all to be from 20th century chemistry, the era of chemistry in which I was an experimental research organic chemist for more than four decades^30−34^ and whose history I have studied since the early 1980s.^35,36^ I wanted to examine candidates that are familiar to many of the current readers of this journal, by virtue of their own professional experiences. I wanted to examine at least one candidate that had previously been declared a revolution in chemistry by multiple scholars, including both historians and chemists.^22^ I wanted to include candidate revolutions from several different subdisciplines of chemistry. I wanted to include one candidate likely to be a revolution in chemistry and another unlikely to be a revolution in science, thereby providing a diversity and range of responses to the definitional interrogation. Last, most of the published studies on revolutions in science and certainly on revolutions in chemistry discuss events prior to the 20th century.^22^ This is likely because most professional historians and philosophers of chemistry, i.e., those with Ph.D. degrees in history or history of science or philosophy, find 20th century chemistry beyond their knowledge base.^37,38^ For all these reasons, I decided to examine 20th century candidates for revolutions in chemistry.

## 3 Are Revolutions in Science Real?

**Figure 1:**
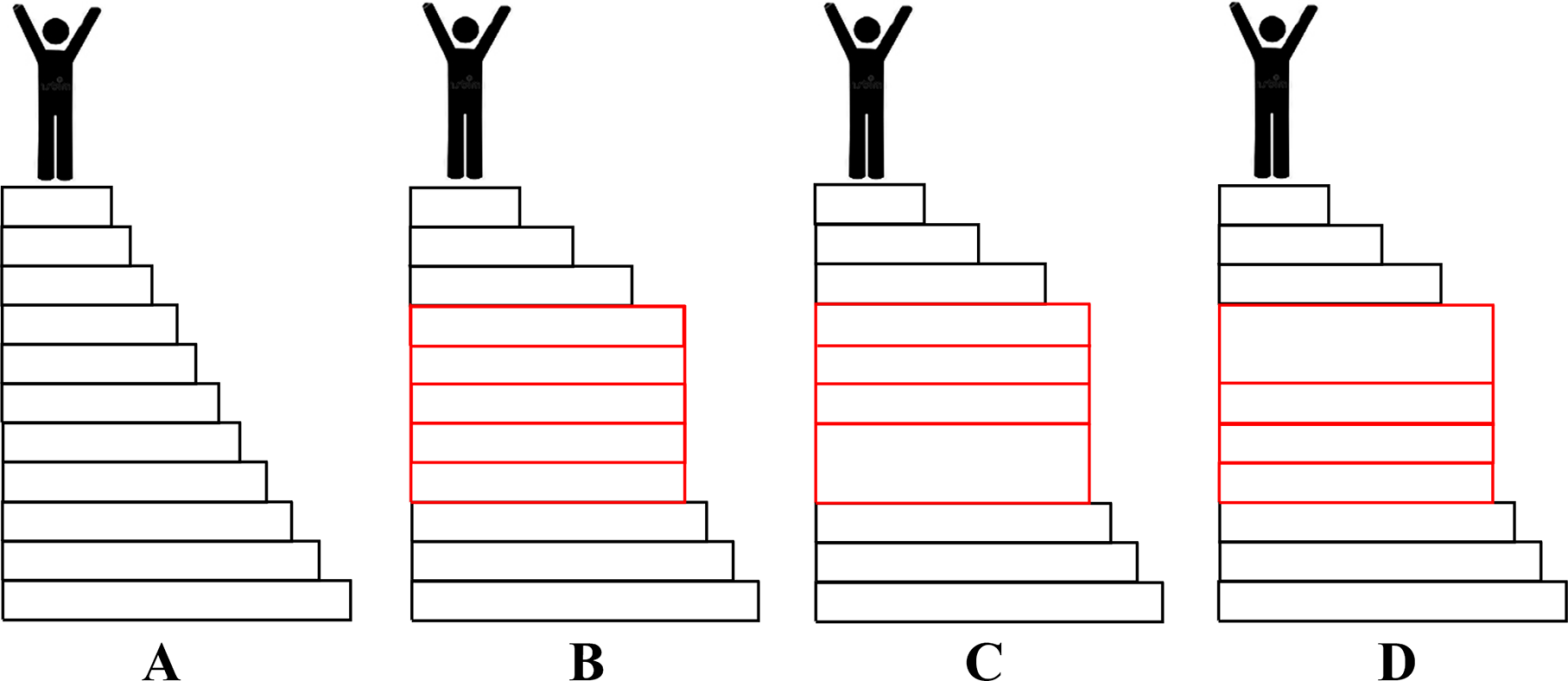
Four illustrations of growth of knowledge. Illustrations B, C, and D represent three types of revolutions in science. The steps represent knowledge growth in science. The individuals at the top step in A–D represent a triumphant scientific community after different sequences of knowledge growth. The smaller steps do not necessarily represent the same magnitude of knowledge growth. There is no meaning to the vertical axis other than to suggest steps in a progression. (A) An illustration of normal science, as first proposed by Thomas Kuhn.^1,39^ (B–D) The disruptive period (the gap jump comprising all the steps in red) indicates a revolution in science, as defined in Table 1. The distinction between B versus C and D is that in the latter two representations, one bigger step functions either as an initiating event (as in C) or as a precipitating event or a tipping point step (as in D). In principle, a revolution in science may include both an initiating step and a precipitating step or tipping point. This graphic is adapted, significantly expanded, and reproduced with permission from ref (22). Copyright 2023 Springer Nature.

Are “revolutions in science” not really revolutions as several historians and philosophers have suggested,^11,25^ just the result of normal progress of science which leads to new knowledge, as illustrated in Figure 1A? Or are there periods of “normal science” punctuated by rare but a series of highly disruptive events that lead to revolutions in science (Figure 1B)? Or perhaps revolutions in science involve a series of small, evolutionary yet scientifically coupled disruptive steps plus one or more initiating or precipitating events which together lead to a revolution in science (Figure 1C or Figure 1D, respectively)?

**Table 1:** Thirteen Characteristics of Revolutions in Chemistry, Collected into Six Factors^a^

		Instrumental revolution						
Characteristics		“Instrumental revolution (1945–1966)”: the literature perspective	Mindful instrumental revolution (this work)	Hückel molecular orbital theory: 1930s (1950s)^b^	Hückel’s 4*n*+2 rule”	Woodward–Hoffmann (W–H) rules	Quantum chemistry	Retro-synthetic analysis
factor 1: causes and birthings of revolutions in chemistry								
1	individual and community-wide disappointments arise with current theories	–	+	– (+)	*m*	+	+	–
2	a state of crisis occurs within the community	–	–	– (−)	–	+	+	–
factor 2: relations between the old and the new								
3	discontinuous characteristics develop during the revolutionary time period	–	+	– (+)	+	+	+	–
4	incommensurability of old and new theories and/or paradigms obtains	–	+	+ (+)	+	+	+	–
5	irreversible changes in individual chemist’s visions and understandings occur^c^	–	+	– (+)	+	+	+	+
6	irreversible community-level change of ideas and shared standards^c^	+	+	– (−)	–	+	+	+
factor 3: Ideational and conceptual qualities of the specific revolution in chemistry								
7	anomalies, disappointments, and crises are resolved with new paradigms and new conceptual developments; solves problems	–	+	– (−)	+	+	+	–
8	irreversible taxonomic and lexiconic changes and characteristics between experimental and theoretical results and explanations^c^	+	+	– (+)	+	+	+	*m*
9	new research problems and programs arise, new phenomena to explain become evident, new ways of seeing the world develop; new predictions are made	+	+	– (+)	+	+	+	+
factor 4: instrumental and methodological functionaries								
10	new instruments and novel aspects of use are invented^c^	+	+	– (−)	–	+	+	+
factor 5: social construction and practical consequences								
11	revolutions in chemistry are analogous to social and cultural revolutions; new organizational structures appear, new subdisciplines are formed, which support the use of the new theories and paradigms	+	+	– (−)	–	+	+	–
12	irreversible educational methods occur^c^	+	+	– (+)	–	+	+	*m*
factor 6: testimonials^d^								
13	testimonials are made by participants, observers, and academics who study such revolutions	+	+	– (−)	–	+	+	*m*
totals								
no. of (full) positive evaluations (13 maximum)		7	12	1 (7)	6 + 1*m*	13	13	4 + 3*m*

a The list of six factors and 13 characteristics with modifications are taken from ref (22). Reproduced with permission from ref (22). Copyright 2023 Springer Nature. The symbols refer to (+) = present and (−) = not present. “*m*” = minor, e.g., the concept of “retrosynthetic analysis” entered into the practice and language of synthetic organic chemists as well as the double arrow to signify retrosynthetic thinking. For the unabridged parent table with detailed descriptions of each of the 13 characteristics along with supporting literature references, see the foundational publication (ref (22)) from 2023.

b Numbers in parentheses refer to the analysis as of the 1950s.

c Modified from the original table, c.f., footnote a.

d “Testimonials” refers to “an expression of appreciation or esteem”; a recommendation by a person or organization.

I suggest that the extensive literature on revolutions in science^22^ written primarily by historians and philosophers of science provides strong *de facto* evidence of the existence of revolutions in science (Figure 1B–D). What do we call periods of science that have few, if any, of the characteristics in Table 1 when compared with other periods of science that are characterized by many if not all characteristics in Table 1 (Figure 1A)? We refer to the latter as “normal” or “evolutionary” science. We refer to the latter as revolutions in science.

In this publication, I shall distinguish Figure 1A periods from Figure 1B–D periods. I shall also distinguish Figure 1B, Figure 1C and Figure 1D periods from each other.

We must discuss chronology as it pertains to revolutions in science. Note that calendrical time is not included in Figure 1, intentionally so. I claim that a revolution in science need not occur within a short time span. (Even political revolutions can occur over several decades, though the tipping points can occur rapidly at the end of the build-up period, as in Figure 1D.) (A “tipping point” is defined as “the point at which a series of small changes or incidents becomes significant enough to cause a larger, more important, and often irreversible change.”^40^)

In discussing multiple simultaneous independent discoveries (and discoveries are always embedded within revolutions in science), the eminent sociologist of science Robert K. Merton discussed the nature of “simultaneity” by distinguishing between “calendrical time” and “social and cultural time.”^41^ Merton wrote:

“The theory does not hold that to be truly independent, multiples must be chronologically simultaneous. This is only the limiting case. Even discoveries far removed from one another in calendrical time may be instructively construed as “simultaneous” or nearly so in social and cultural time, depending upon the accumulated state of knowledge in the several cultures and the structures of the several societies in which they appear.”^41^

I claim that “calendrical time” is not a controlling factor in assessing revolutions in science. But if “rapid change” is not a mandatory characteristic of revolutions in science, what might be? I posit that the characteristics in Table 1 are the mandatory characteristics, or at least some combination of those characteristics. As discussed below, I believe there are intellectual and mindful requirements as well as intimate involvement of social issues, propelling the revolutions in science and resulting from the revolutions in science. I also conclude that elements of social construction of knowledge^42−45^ are essential within revolutions in science. In these analyses, I very much favor Merton’s “cultural time”.^41^

## 4 Candidate 1: the “Instrumental Revolution”

### 4.1 Background: The Evidence

Many publications in the history and philosophy of chemistry have identified the “Instrumental Revolution” as having occurred in the period following World War II and lasting about two decades.^14,18,46−67^ In 2019, José Chamizo, who has published extensively on revolutions in chemistry,^14,18,64,66^ postulated the dates 1945–1966 for the Instrumental Revolution and designated it to be “the fourth chemical revolution.”^14^

In his *Preface* to the 19-chapter, multiauthored book entitled *From Classical to Modern Chemistry: The Instrumental Revolution,*(8) the highly acclaimed, self-identified “structure elucidation chemist”^46^ Carl Djerassi, noted for his hundreds of publications^68−70^ on mass spectrometry, optical rotation (CD and ORD), and magnetic circular dichroism, said,

“The papers in this volume are of interest to me, because I took part in the “instrumental revolution” myself. [The essays therein] demonstrate the enormous impact that new physical instrumentation had on chemistry and biochemistry.”^46^

**Figure 2:**
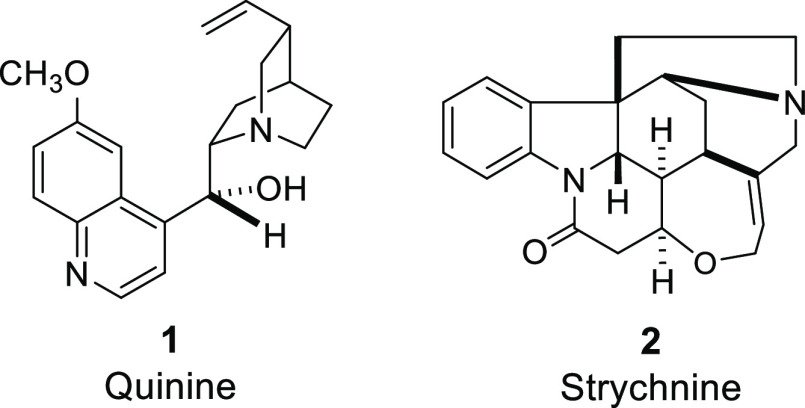
Structures of quinine (**1**)^75−77^ an strychnine (**2**),^78−82^ two complex natural products whose structures were determined primarily using the classical methods of structure determination.^83,84^

For chemists, two key tasks were and are purification and structure identification of natural products and newly synthesized materials. Chromatography transformed chemistry.^71^ Previous to chromatography, the typical methods of purification were fractional distillation and crystallization. For alkaloids that were oils, that meant preparation and purification via selective crystallization of, e.g., their salts, e.g., tartrates. A classic in the history of purification of natural products by crystallization is that of the purification of the diastereomers (excluding enantiomers) of quinine (**1**) (Figure 2) via their acid salts by Paul Rabe in the first quarter of the 20th century.^72−75^

The 1940s and 1950s indeed began an era of new instruments, new methodologies, and new types of data and a host of new types of research programs.^8,85−88^ And perhaps, given the discussion above about “chronological time” and “conceptual time,” the time period for the Instrumental Revolution ought to be increased on both ends of the scale, beginning in the 1940s and extending into the 1970s. This is just a numerical detail, not a conceptual consideration.

### 4.2 Was the “Instrumental Revolution” A Revolution?

Was there a crisis, using Kuhn’s terminology,^39^ for chemists, that purification and identification of novel compounds was in such a primitive state prior to the 1940s? Chemistry, i.e., structures, research programs, and physical and chemical properties being examined, were getting more and more complex. The nature of drug discovery and synthesis required purity and analyses that simply were not possible when “our most versatile organic laboratory instrument”^89^ was the thermometer, as stated by the NMR guru of the 1950s and 1960s, John D. Roberts.

Progress in chemistry required efficient and speedy structure identification. Forty-year periods for structure determinations as for strychnine (**2**, Figure 2) seriously delayed progress in chemistry. In terms of counterfactual history of chemistry,^90,91^ in the absence of the growth of instrumental analysis and modern purification techniques, I believe that resources would not have continued to flow into chemistry had its rate of progress been that of even the 1940s while other sciences were roaring along. Chemistry would have become a much slower growing science.

But was there a crisis brought about by what we know today as seriously inefficient structure determinations prior to the era of spectroscopy? I do not believe there was such a crisis.

There can be no disagreement that the invention and commercialization of instrumentation was transformative in the practice of 20th century chemistry (and other disciplines as well, especially the life sciences). The speed of purification and identification of new compounds increased by many orders of magnitude. New physical properties of compounds were identified, measured, and archived. But what about the claim that this was an “Instrumental Revolution”? As mentioned at the top of this section, there is today and in the past 25 years near consensus within the history and philosophy of chemistry literature that there was an instrumental revolution. But my evaluation and analyses of the “Instrumental Revolution” (see the evaluations in Table 1) suggest a more nuanced situation. Chemists of that era did their chemistry without any sign of crises caused by experimental inefficiencies; they knew no better. They had no anticipation of what the future would bring in terms of analytical instrumentation, and thus they were not frustrated by what was the norm of the era.

As described in the philosophy of chemistry literature, their “Instrumental Revolution” (i) lacks the intellectual component of a revolution in chemistry, (ii) did not consider the massive amount of data provided by this growth in instrumentation and its synergistic power, (iii) did not include any community-wide disappointments in current theories nor was it preceded by a deep crisis, and (iv) did not involve any incommensurability of competing paradigms or involve two opposite (or several differing) points of view or some type of conceptual dissonance that was resolved by a paradigm shift. This analysis is reflected in the assignment of seven “checks” in Table 1 for the literature perspective of the Instrumental Revolution.

Several historians and philosophers of chemistry have previously noted the lack of these characteristics in the “Instrumental Revolution” but had not considered those characteristics to be disqualifications for a revolution in chemistry.^14,18,49,88,92−94^ Indeed, some scholars have directly faced the issue as to whether intellectual and social construction of science components are necessary for a revolution and have used the invention of tools as a counter example; they have concluded that tools are sufficient.^95,96^ My analysis (Table 1) suggests otherwise, that an intellectual component beyond the invention of tools is necessary for a revolution in chemistry.

### 4.3 The “Mindful Instrumental Revolution”

I now explore a much expanded and nuanced view of the “Instrumental Revolution.” I shall provide several aspects of the invention and use of instruments that support the “Mindful Instrumental Revolution” as being a revolution in chemistry.

#### 4.3.1 Going Beyond Structure Determinations

In and subsequent to the mid-1940s, there was an expansive intellectual drive for new instrumentation that went far beyond structure determination. There were chemists who needed new instruments for their own unique reasons, not just for structure determination. They wanted to study very complex physical and eventually chemical properties of matter. These were physical chemists and chemical physicists who were inventing instruments to solve specific research problems, not for commercialization and sale to a hungry worldwide community eager to solve structural questions more easily. Think of molecular beam studies and nano, even femtosecond spectroscopy, super-resolved fluorescence microscopy, scanning tunneling and electron microscopy, soft desorption ionization methods, and so on.

Several excerpts from Nobel Prize Lectures of inventors of instruments are instructive. These two excerpts highlight the intellectual ingredients required for the mindful invention of an instrument: the recognition of a specific research need, the design and construction of prototype instruments, the testing and their use of the instrument in research programs, and perhaps even the eventual commercialization of such an instrument.

(i) From Jaroslav Heyrovský’s 1959 Nobel Prize lecture “for his discovery and development of the polarographic methods of analysis”: “To accelerate the plotting of the curves we have constructed with [Masuzo] Shikata in 1924 an automatic device, the “polarograph” by rotating the Kohlrausch drum mechanically, and synchronously moving also a photographic paper... ”^97^
(ii) From Aaron Klug’s 1982 Nobel Prize lecture “for his development of crystallographic electron microscopy...”: “The formidable technical problems were overcome only after the development in our laboratory of more powerful X-ray tubes and of special apparatus (cameras, computer-linked densitometers) for data collection from a structure of this magnitude. (In fact we had begun building better X-ray tubes in London to use on weakly diffracting objects like viruses).”^98^

Similar stories by the inventors of their inventions can be found in the Nobel Prize lectures of Otto Stern (1943), Isidor Rabi (1944), Felix Bloch and Edward Mills Purcell (1952), Richard Ernst (1991), Kurt Wüthrich (2002), and Paul Lauterbur (2003). Other such intellectually oriented recollections can be found in autobiographical essays of other instrument inventors as well.

The point is, there were and are many mindful, intellectually laden steps in the invention of new instruments: the identification of and decision to obtain specific types of data for a clearly specified objective; the design and testing and redesign of the prototype instrument; actual experiments to obtain data; and analysis of the data, conclusions, and theory redefinition. Of course, mindfulness is a characteristic of any good scientific study.

I hasten to add: the Mindful Instrumental Revolution was accompanied by and facilitated by significant irreversible changes in laboratory practice. A recently published book on this topic with contributions by numerous scholars in the history of chemistry covers much of this territory (*The Laboratory Revolution and the Creation of the Modern University, 1830–1940*^99^). This volume follows closely behind another book by the historian of chemistry Peter J. T. Morris, *The Matter Factory: A History of the Chemical Laboratory*.^86^

#### 4.3.2 Instrument-Based Chemists and Chemistry

Many of the characteristics of a “revolution in chemistry” in Table 1 require a breadth and magnitude of effect on the discipline. Note such words in Table 1 as community wide, crisis, catastrophic rupture, transformation from a mature science, and indeed, the terms revolution and revolutionary divide. It stands to reason that not every major scientific breakthrough, indeed, not even every Nobel Prize, could initiate a revolution in chemistry. The proponents of the “Instrumental Revolution” have suggested that it was the methodological efficiencies of the newly commercialized instruments in the 1945–1966 era that transformed chemistry. Examples cited were the ability to determine the structure of natural products in days rather than in years or decades, as in the X-ray structure determination of strychnine.

I posit that the mindfulness of the instrumental revolution may be with the people who invented the instruments and with the people who actually used them. As mentioned above, many instruments have been invented because a researcher wanted to come up with a better or new way of making a certain measurement or they want to exploit a fundamental physical or chemical property. This is as mindful as the actual applications, even if many of the applications are devised far later than the invention and were never anticipated by the inventors.

In terms of the social construction of knowledge, the advent of “big instruments” was accompanied by physical chemists and chemical physicists who designed, built, operated, and did research with these instruments. Plentiful governmental funds were available during World War II and thereafter to fund the development of new instrumentation, in particular, and science, in general.

#### 4.3.3 The Massive Quantity of Analytical Data

There is another very important consequence of all these new instruments: the sheer quantity of information. The increased funding for research by national governments^100^ and the expanded emphasis on research at universities and colleges, plus the heightened efficiency of doing chemical research, in large measure due to the introduction of new instrumentation, created a magnified, indeed accelerated quantity of chemical knowledge that derived from the instrumental analyses. Furthermore, with this new knowledge and these new instruments came new ways to carry out chemical research. This explosion of data and knowledge was accompanied by various types of new connections: connections between people and connections between molecules, reactions, and physical properties. The realization of these synergies at a magnitude never before seen was the second type of intellectual outcome related to an instrumental revolution in chemistry. The result was an extraordinary outpouring of new ideas, new problems, and new knowledge.

#### 4.3.4 New Scientific Questions and Programs That Were Spawned from the New Instruments

New instrumentation led to scientific questions never previously anticipated, even in the minds of the inventors and designers of this instrumentation, and even in the minds of the most forward-thinking and imaginative chemists. Novel aspects of use of already designed instrumentation provided further opportunities for new scientific questions and programs. For example, in 1968, Gerhard Binsch wrote a 54-page chapter in *Topics in Stereochemistry* on “The Study of Intramolecular Rate Processes by Dynamic Nuclear Magnetic Resonance,”^101^ a topic that became evident only after the instrumental advances that allowed for its investigation. As a second example of new research questions following the development of new instrumentation, Jack Dunitz showed that X-ray crystallography could extend far beyond its typical challenge of structure determination. Dunitz’ series of studies on “Chemical Reaction Paths”^102^ is just one theme that X-ray crystallography addressed that also exemplifies this theme. Instrumentation not only solved old problems, it also provided the ability to create new research directions.

#### 4.3.5 Putting It All Together: “The Mindful Instrumental Revolution”

It is evident that the Instrumental Revolution is meant both in the service of instrumental techniques as tools of chemical analysis and as a possibility to learn about the nature of chemical bonding and reactivity in its broadest interpretations.

With these added insights, the intellectual components of instrument design and implementation, the exponential increase in the amount of new knowledge, and the modern methods of envisioning, selecting, and performing new projects, I revisited Table 1 with expanded understanding of a “Mindful Instrumental Revolution” and ultimately judged this to be a revolution in chemistry. Compare columns 2 and 3 in Table 1. Based on a minfulness component, I judged the Industrial Revolution to have 12 “checks” in Table 1.

When we think of the Instrumental Revolution in 20th century chemistry, we recall that commercial infrared spectrometers, NMR spectrometers, mass spectrometers, and ultraviolet spectrometers did not arrive simultaneously on the commercial scene. There was perhaps a 30-year gap between the earliest and the last of these as user-friendly instruments. Figure 1B seems to be the best illustration of this revolution in chemistry. I cannot identify any particular instrument that served either as an initiator (Figure 1C) or as a precipitator (Figure 1D) of the Instrumental Revolution of the 20th century.

A very recent abstract for an upcoming Annual Meeting of the Japanese Society for the History of Chemistry (July 8–9, 2023) is entitled “On the Instrumental Revolution in Chemistry. Retrospective and Prospect” by Mari Yamaguchi.^67^ I am confident that this lecture, when it becomes available in a journal article, will add to the assessment and analysis of the Instrumental Revolution.

### 4.4 Unanticipated Value of This Type of Analysis. Part 1

I must acknowledge the process that led to my reassignment of the instrumental revolution. My initial application of the criteria for the “Instrumental Revolution” (Table 1, column 2) led to a negative classification. This did not match the literature attributions of many historians and philosophers of science and the instincts of practicing chemists including one of my own heroes, Carl Djerassi. I pondered. I rethought the analysis.

I then focused on what was being undervalued in my initial analysis of the “Instrumental Revolution” (Table 1, column 2). I realized that I needed to take into consideration the ideas and motivations of and the risks undertaken by many intellectually engaged scientists in their conception, design, testing, use, and introduction of new instruments. I also needed to consider the effects of all these instruments on the much-enlarged body of new data obtained by and communicated within the community. This resulted in a better tracing of the intellectual effects of instrumental advances on the rest of chemistry. An updated evaluation (Table 1, column 3) resulted. With the revised understanding of the “Mindful Instrumental Revolution” but still using the same criteria in Table 1, I concluded that there was an instrumental revolution in the second half of the 20th century. And my updated conclusion was now in synch with the literature analyses of many historians and philosophers of chemistry.

An anonymous reviewer asked, “If you had not been able to go back and find evidence that the ‘Instrumental Revolution’ could be made to fit your definition of a revolution, would that have made you rethink your definition/criteria?” I believe the answer to that question is “yes, rethink” but “no, not necessarily change my decision.” My esteem for those who preceded my analysis is such that I would have had to reexamine the definition/criteria several times before concluding that earlier scholars were wrong.

Nonetheless, these considerations reinforce my feeling that definitions ought not necessarily be set in concrete. With the benefit of time and additional knowledge and experiences, mindful reviews of definitions may well lead to appropriate revisions of Table 1.

## 5 Candidate 2: Hückel MO Theory

The second of the six candidates being examined as a revolution in science, Hückel molecular orbital theory (HMO theory), was chosen for several cooperative reasons: (i) because it was the first quantum chemistry theory that was the starting point for teaching quantum chemistry to experimental chemists, especially organic chemists,^103−106^ (ii) because it was the springboard into the quantum chemical analysis of complex reactions, and (iii) because HMO theory served as the basis upon which another theory was developed. That other theory plays a major role in this discussion. It was extended Hückel theory (eHT) and was developed by Roald Hoffmann and other members of William Lipscomb’s research group at Harvard in the early 1960s.^107,108^

In the 1920s and 1930s, as novel ideas about chemical structure and bonding were being formalized by Gilbert N. Lewis, Irving Langmuir, Linus Pauling, and Robert S. Mulliken, among others, more complex compounds were being identified and synthesized, and the first electronic theory of organic chemistry was developed.^109,110^ Chemists were using chemical structures with greater confidence and were developing more complex structure–reactivity properties. Molecular orbital (MO) theory^111^ and valence bond theory^112−114^ can trace their beginnings to this time period. Intuitive heuristic models, like Robert Robinson’s use of curly arrows to describe electron flow in reactions,^115^ were discovered in these years.

In a series of papers first published in 1931, Erich Hückel made a leap in theory by separating σ and π bonding and treating the latter using simple MO theory.^116−120^ Hückel’s molecular orbital (HMO) theory allows the calculation of the stabilization energies and other properties of planar cyclic aromatic compounds and acyclic polyenes. But no one paid much attention to HMO theory for several decades, an example of a sleeping beauty in science, i.e., a publication whose importance is not recognized for more than several years after its original publication.^121,122^ On this basis, judged from the perspective of the 1930s, I rated HMO theory as not causing or initiating a revolution in chemistry (column 4 in Table 1, one check).

But what about the consequences of time? By the late 1940s and through the 1950s, a small number of highly active chemists, mostly but not entirely organic chemists, used HMO to explain the reactivity of simple aromatic compounds. For example, Michael J. S. Dewar calculated the resonance energies of various aromatic systems and by HMO.^123^ In 1947, Dewar calculated the heats of formation of π-complexes using HMO.^124^ In 1952, Kurt Mislow used HMO theory to calculate the “aromatic character” of a series of C_*n*_H_*n*_ monocycles where *n* is even and greater than 8).^125^ Many other examples of the use of HMO theory in this era were reported in Andrew Streitwieser’s 1961 textbook on *Molecular Orbital Theory for Organic Chemists*.^104^

But the value of HMO theory were time-limited for several reasons. First, as John Pople stated in 1953, “Although it has the merit of great simplicity, the Hückel procedure has serious defects. These are connected with ... [its simplicity!]”^126^ Pople then continued with an early yet detailed explanation of HMO theory’s defects. Not emphasized by Pople but critical to those interested in using theory on complex molecules: HMO theory’s calculations were limited to planar systems in which the σ-bonds were essentially ignored. Pople, Dewar, Hoffmann and Lipscomb’s research group, Kenichi Fukui, and many others began to develop more complex and expansive MO theories.

**Figure 3:**
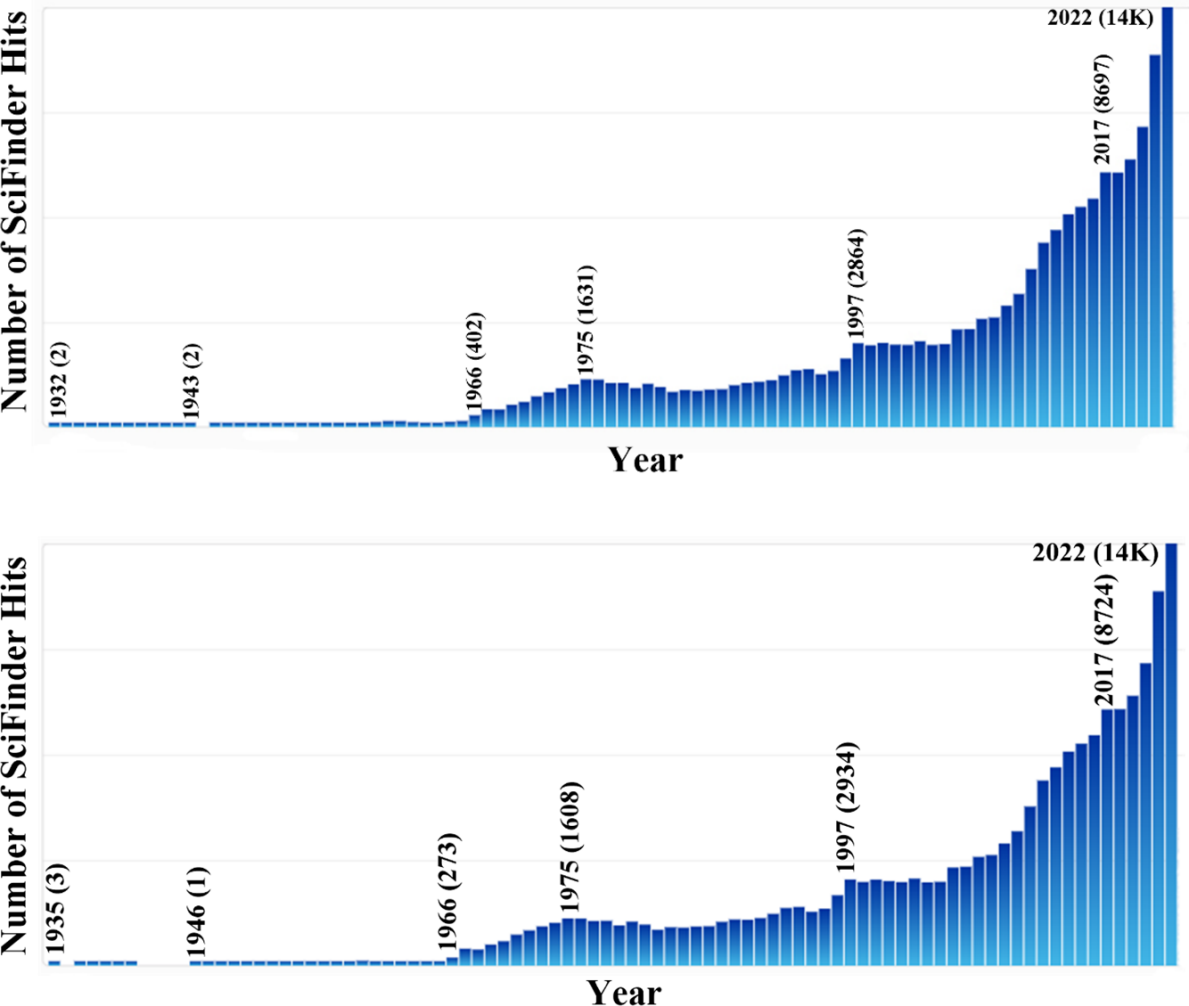
Number of SciFinder^n^ hits for (top) “mol. orbital” and (bottom) “molecular orbital” from 1932 to 2022. “Hits” refers to the number of publications in which the search term is found at least once in either the publication’s title, abstract, or concepts list. For both of these searches, the rise begins in the mid-1960s and continues with an acceleration in the late 1990s. The first 10 hits for “molecular orbital” include a who’s who of quantum chemistry: Robert S. Mulliken (3 times), Charles A. Coulson (3 times), John E. Lennard-Jones (once), and George W. Wheland (once).

By the 1960s, molecular orbital theory had entered the world of experimental chemists, notably also organic chemists, as can be seen from the SciFinder^n^ hits shown in Figure 3 for “molecular orbital”. The term “molecular orbital” (MO) first appears in the SciFinder^n^ search with 1933 publications by Mulliken,^127−129^ by John E. Lennard-Jones,^130^ and by Charles Coulson including coauthors H. Christopher Longuet-Higgins^131−133^ and Dewar,^134^ and a 1938 singly authored paper by George Wheland.^135^ Not surprisingly, these were the premier theoretical chemists of the era, two of whom, Longuet-Higgins^136^ and Dewar,^137−139^ made seminal contributions to the Woodward–Hoffmann (W–H) rules. There are bibliographic data illustrating the rapid acceleration of “molecular orbital(s)” beginning in the 1960s (see Figure 3).

It may well be convincingly argued that HMO theory is one of the tangible and lasting pioneering achievements of early quantum chemistry, perhaps in the guise of pedagogy, perhaps in its continued though infrequent use in the guise of extended Hückel theory, or perhaps simply because of its place in the history of chemical theory. By and large, HMO theory slept through most of the 1930s and 1940s. Why? One reason, because HMO theory was a German discovery, published primarily in German physics journals.^116,118−120,140−143^ The eventual practitioners of HMO theory were physical organic chemists. And in that era, physical organic chemistry in Germany was a subdued if not an unemployed discipline.^144^

Furthermore, to take HMO theory to a quantitative level, it needed to wait until the availability of even rudimentary computer power. That would not happen until after World War II. Even Walter Hückel, one of the few German physical organic chemists in the first half of the 20th century, almost completely excluded molecular orbital theory and even his own brother’s HMO theory from his (Walter Hückel’s) multiedition multivolume textbook *Theoretical Principles of Organic Chemistry*.^145^ Another reason, the application of HMO theory beyond qualitative analysis awaited the development and commercialization of computers. And third, physical organic chemistry as a subdiscipline of chemistry was still very much in its infancy, as was organic chemistry. An eruption of chemical knowledge would come after World War II.

With this background, using the criteria in Table 1, let us interrogate HMO theory as initiating a revolution in chemistry. From the perspective of the 1950s, HMO theory did not, nor did any other aspect of molecular orbital theory, initiate a revolution in chemistry (column 4 (in parentheses) in Table 1, seven “checks”). In that time period, no crisis in chemistry was resolved, and there was no community-wide change of ideas or shared knowledge due to HMO theory. No new subdisciplines of chemistry were formed. No new journals appeared. No new specializations of academic research groups or industrial organizations resulted thereof. No new educational methods were adopted, other than another set of equations. In a sense, Figure 3 tells it all. There was no intense use of any MO theory including HMO prior to the 1950s, if not until the mid-1960s. I therefore conclude that HMO theory was not a revolution in chemistry, nor did it initiate one.

## 6 Candidate 3: Hückel’s Rule

The third of the six candidates being examined as a revolution in science, “Hückel’s rule (4*n* + 2) for aromaticity”, was chosen because it was one of the first quantum chemistry rules dealing with the relationship of structure with both physical and chemical properties that remains relevant and valued today. In addition, Hückel’s rule, first developed in the early 1930s^116,117,119,140,141^ though not stated explicitly in this simple and easily identified and used equation-like form until 1951 by William von E. Doering and Frances L. Detert,^146^ could have, but did not, initiate a demonstrable interest among organic chemists in molecular orbital theory as did the W–H rules a decade later (candidate 4).

Hückel’s early publications on HMO theory calculated the “resonance energy in [the] ground state” of acyclic polyenes and monocyclic and several polycyclic aromatic compounds including cyclobutadiene, benzene, and cyclooctatetraene.^117^ He then explained the differences in stability using his simple yet elegant and breakthrough MO theory. But it was not until the 4*n* + 2 formula was proposed^146^ that one of organic chemistry’s most treasured and cited “rules” for predicting stability and reactivity of aromatic and antiaromatic compounds became used. HMO theory was also used in the 1950s and early 1960s to calculate reactivity indices for aromatic substitution reactions.^104,147^ Especially notable is that the early career of Ronald Breslow was centered around examining rather unique monocyclic compounds having 4*n* + 2 π-electrons, i.e., aromatic character, and 4*n* compounds having antiaromatic character.^148−152^

Did Hückel’s rule initiate a revolution in chemistry? My analysis says “no” (see Table 1, column 5; six checks plus one minor check). There were simply no broad community-wide disappointments prior to Hückel’s rule and certainly no state of crisis. Chemists were content in rationalizing the stability of benzene, typically by Crocker’s and Robinson’s rule of six,^153−156^ and they were satisfied to explain the inability to synthesize cyclobutadiene due to the strain of including two double bonds in a four-membered ring. Hückel’s rule did not remove any widespread disappointment with existing practices or existing theories, nor did it resolve a catastrophic rupture in chemistry or replace any faulty concept that was of community-wide concern. The promulgation of Hückel’s rule did not undermine any shared standards of the research community (whether or not Hückel’s rule included the “4*n*” component). There was no social breakdown or reorganization of the structures of chemistry due to the introduction and promulgation of Hückel’s rule. Scientists using Hückel’s rule certainly did not think of themselves as revolutionary nor have subsequent scientists or historians of chemistry thought so. Neither historians nor philosophers of chemistry considered Hückel’s rule as initiating a revolution in chemistry.

Furthermore, Hückel’s rule did not initiate a community-wide acceptance and use of computational chemistry as did the Woodward–Hoffmann rules (discussed in the next section). Indeed, Hückel’s rule was of very limited scope and was narrow in its influence. What Hückel’s rule did not do that Woodward–Hoffmann rules did do was to create a new subdiscipline of chemistry along with an ever-growing army of computational chemists, transformations that continue today, many decades later. It is true that R. B. Woodward and Hoffmann did their research in the mid-1960s, when computer hardware, at least on the level usable for Hoffmann’s extended Hückel program, was available. Hückel and his rule were sleeping beauties, an unfairness of life. This is an example of the social construction of knowledge: no computers, no revolution; less relevant chemistry, no need for the rule.

Nevertheless, Hückel’s rule did have six-plus characteristics of a revolution in chemistry, confirming that Hückel’s rule was a major breakthrough in chemical thought and practice. It just was not a revolution in chemistry, nor did it initiate or precipitate one.

## 7 Candidate 4: The Woodward-Hoffmann Rules (The Principle of Conservation of Orbital Symmetry)

### 7.1 Background: The No-Mechanism Problem

The fourth of the six candidates examined herein as revolutions in chemistry is the principle of conservation of orbital symmetry, more commonly referred to as the Woodward–Hoffmann rules.^157−159^

By the early 1960s, chemists had formulated many heuristic theories with which they explained the physical and chemical properties of all chemistry. Heuristic theories and reasoning by analogy are based on an ever-expanding set of experimental data using intuition and developing simple models, e.g., Robinson’s and Christopher K. Ingold’s electronic theory of organic chemistry, steric hindrance, conformational analysis, and especially arrow pushing.^29^ Heuristic theories, sometimes known as “soft theories,” are mostly qualitative, though some have quantitative aspects to them, e.g., the (Louis P.) Hammett equation.^160−162^

In the early 1960s, Doering gave the name “no-mechanism” to reactions that were then known as valence isomerizations and in some instances tautomerization for which there was no known mechanism. Doering asserted that

““No-mechanism” is the designation given, half in jest, half in desperation, to “thermo-reorganization” reactions like the Diels-Alder and the Claisen and Cope rearrangements in which modern, mechanistic scrutiny discloses insensitivity to catalysis, little response to changes in medium and no involvement of common intermediates, such as carbanions, free radicals, carbonium ions and carbenes.”^163^

**Figure 4:**
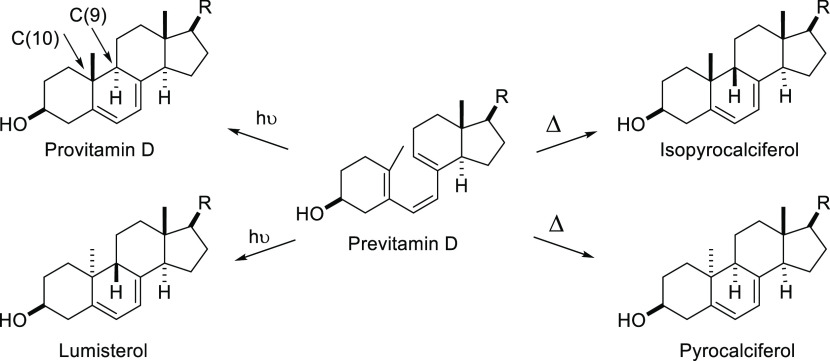
Two sets of stereospecific reactions in the vitamin D series observed and discussed by Egbert Havinga and his group and Luitzen Oosterhoff at the University of Leiden in the early 1960s.^164^ The puzzle was why certain reactions proceeded thermally and others photochemically and why the stereochemical preferences at C(9) and C(10) in these reactions are as shown in this graphic. “R” represents the cholesterol side chain.

Before the W–H rules, there was no explanation (i) why [2 + 2] dimerizations of olefins typically occur photochemically and not thermally (except when the olefins are highly substituted with very powerful electron donors or acceptors); (ii) why [4 + 2] cycloadditions, e.g., the Diels–Alder reactions, occur easily under thermal conditions (all suprafacial additions); why thermal 1,3-hydrogen migrations are rare yet thermal 1,5-hydrogen migrations are frequent; (iv) why some 1,3,5-hexatrienes thermally close to 1,3-cyclohexadienes of certain stereochemical configurations but ring close to the opposite configuration upon photochemical conditions (Figure 4); and why *cis*-3,4-disubstituted cyclobutenes thermally ring open to the less stable *E,Z-*1,3-butadienes.

There is no “soft theory and reasoning by analogy” solution to the no-mechanism reaction. None. Whether arrow pushing and reasoning by analogy, steric effects, stereoelectronic effects, and conformational analysis, none of those heuristic models or any combination of them could (or can) explain the no-mechanism problem. An entirely new type of mechanism was needed though that was not obvious at the time to the practitioners.^165−168^ And that mechanistic requirement was an application of quantum chemistry.

### 7.2 The Solution to the No-Mechanism Problem: The Woodward–Hoffmann Rules

Beginning in January 1965, Woodward and Hoffmann published a series of five communications in the *Journal of the American Chemical Society* (*JACS*) that provided the mechanism of the no-mechanism problem.^169−173^ Today we know their solution as the Woodward–Hoffmann rules. Woodward and Hoffmann used a variety of MO tools to explain what they called pericyclic reactions: electrocyclizations, cycloadditions, sigmatropic rearrangements, cheletropic rearrangements, and group transfers and eliminations. Those MO tools included frontier MO theory, calculation of simple potential energy surfaces using extended Hückel theory, perturbation theory, and correlation diagrams and interaction diagrams.

### 7.3 Did the Woodward–Hoffmann Rules Initiate or Precipitate a Revolution in Chemistry?

I posit that the W–H rules initiated a revolution in chemistry. No longer would “heuristic models and reasoning by analogy”^29^ be the final word in the explanation of chemical phenomena. But I conclude this, not solely or even primarily for the following three reasons, all of which are true: (i) The Woodward and Hoffmann rules provided the mechanisms of pericyclic reactions and, in doing so, cleared up a considerable void in chemical knowledge, i.e., the W–H rules explained and predicted. (ii) The W–H rules were both portable and expandable. Scholars proposed variations of the original rules and new concepts in chemistry and simultaneously proposed a number of new terms, e.g., enzymatic pericyclic reactions^174,175^ and mechanochemical pericyclic reactions.^176^ (iii) The scientific community certified the importance of the Woodward–Hoffmann rules by (a) the award of the 1981 Nobel Prize in chemistry to Hoffmann, (b) by the inclusion of this concept in perhaps every undergraduate organic chemistry textbook, and (c) by its being used in today’s research as a tool in synthesis and as an object of continuing study.

Well beyond the above achievements, as a result of my interrogation of the 13 characteristics and six factors of revolutions in science (column 6 in Table 1, 13 checks), I conclude that the W–H rules did precipitate a revolution in chemistry. In particular: (i) The Woodward–Hoffmann rules were the first demonstration to a wide body of chemists that quantum chemistry was an absolute necessary and integral explanatory component for most chemistry. (ii) Woodward and Hoffmann established that the collaboration of an experimental chemist with a computational chemist produced a synergy of inestimable value. The synergy obtained through such a collaboration could be both problem-solving, research program-generating, and career-determining. (iii) The influx of applications of quantum chemistry led many would-be chemists to become computational chemists. Many synthetic chemical research groups now include computational chemistry as a functioning tool if not computational chemists themselves. (iv) Ultimately, W–H led to the formation of a new subdiscipline of chemistry, i.e., computational chemistry. New journals and textbooks of computational chemistry appeared. Research departments of computational chemistry were initiated, especially in the pharmaceutical and chemical industries. As Herbert Mayr said in a 2016 review of physical organic chemistry,

“Nowadays, quantum chemical calculations have reached such a level of accuracy that some journals are already reluctant to publish mechanistic proposals without computational support.”^177^

Bibliographic analysis supports the conclusion that the W–H rules initiated a revolution in chemistry. Note the steep rise in the use of the term “molecular orbital” in the title, abstract, or concepts list of publications within the SciFinder^n^ database (Figure 3) just in the years after the W–H rules were published. But were the Woodward–Hoffmann rules in and of themselves a revolution in chemistry? I conclude otherwise. The next section will place the Woodward–Hoffmann rules within the broader context of quantum chemistry.

## 8 Candidate 5: The Quantum Chemical Revolution

Interrogation of the characteristics of revolutions in chemistry in Table 1 demonstrates that “quantum chemistry” as a chemical theory and an intellectual tool had significant, indeed revolutionary effects on the science of chemistry (13 checks in column 7 in Table 1). Quantum chemistry also influenced the organization and social construction of chemistry. The beginnings of quantum chemistry can be traced to the late 1920s, and the research of Walter Heitler and Fritz London followed shortly thereafter by contributions from Robert S. Mulliken, Max Born, Linus Pauling, Vladimir Fock, Linus Pauling, Erich Hückel, among others. These achievements can be culled together as initiators of the Quantum Chemical Revolution (Figure 1C). In due course, the quantum chemical revolution incorporated Hückel’s rule and later the Woodward–Hoffmann rules. I posit that the W–H rules were the precipitating event or the tipping point that culminated in many of the “plus” judgements in Table 1, especially those dealing with the social construction of chemical knowledge (Figure 1D).

Let us step back in time from the mid-1960s and provide some historical details to this conclusion. In its earliest applications, quantum chemistry helped explain aspects of spectroscopy,^178−180^ the development of the transition state model of chemical reactions,^181,182^ and many other theories, e.g., the Kirkwood-Westheimer theory of electrostatic effects on acid dissociation^183,184^ as well as the Debye–Hückel theory,^185,186^ which was an important step in treating ionic solutions and inorganic chemistry. MO theory was first advanced, as described above, in the late 1920s and early 1930s^179,180,187−189^ and, together with valence bond theory^190,191^ born in the same time period, eventually became the basis of computational chemistry. The James-Cooledge treatment of the wave function and their calculations in 1933^192^ on simple molecules were extended in the early 1960s by Clemens C. J. Roothaan^193^ and Mulliken.^194−196^ In 1943 and 1945, Longuet-Higgins and Ronald P. Bell explained the structure of diborane using quantum chemistry.^197,198^ In the 1950s, the Walsh diagrams provided the potential energy of a molecule as a function of certain conformational changes.^199,200^ In the late 1940s and early 1950s, a handful of chemists, most notably Fukui, Dewar, and Streitwieser were using MO theory to derive reactivity indices and other physical and chemical properties for planar aromatic compounds.^201−206^ Perturbation theory proposed by Dewar in 1952 gave insights into structural and reaction mechanisms and energetics.^203,207−211^ In 1953, William Moffitt and, independently, Jack Dunitz and Leslie Orgel^212^ explained the stability of ferrocene using simple MO theory. Others using quantum chemistry were Pople,^213,214^ Ken’ichi Fukui,^215−219^ Lionel Salem^27,220^ and, in uses beyond perturbation theory, Dewar.^221−224^ Indeed, all of quantum mechanics goes back to understanding the particle-wave duality.

Perhaps it was the organic chemists who were uninvolved in the first 30 years of quantum chemical applications in their research, with notable exceptions. But for the reason now identified,^167,168,225^ those notable exceptions, Dewar being one, did not bring their colleagues into quantum chemistry.

With the above examples in hand, including the W–H rules, I applied Table 1 to the candidacy of the “Quantum Chemical Revolution.” See Table 1, column 7 (13 checks). I conclude in the affirmative, that there was a Quantum Chemical Revolution in the 20th century. This conclusion places the W–H rules as a component, indeed, a tipping point component, in the Quantum Chemical Revolution (Figure 1D).

But here, we run into two serious challenges to this conclusion. First, must revolutions in chemistry happen in a chronologically short period of time? The graphics in Figure 1 do not include any indication of time. It makes intuitive sense that revolutions in chemistry ought to be quick and dramatic, thus favoring “The Woodward-Hoffmann Revolution” when compared to the “Quantum Chemical Revolution.” On the other hand, the W–H rules were a singular achievement within the bailiwick of quantum chemistry. I tend to agree with Merton and favor “cultural time” over “chronological time.”^41^

Second, quantum chemistry in the 1930s and 1940s to the mid-1960s was not called upon as a standard (orthodox, “go-to”) tool to solve complex problems in chemistry by organic chemists. During that time period, physical chemists, including spectroscopists, and theoretical chemists certainly used quantum chemistry in their research. But the use of quantum chemistry to explain chemical reactions was lacking. One major example: the solution to the no-mechanism problem discussed in section 7.1 was not immediately forthcoming. Even those organic chemists who knew of the no-mechanism problem and also knew some measure of quantum chemistry, even those chemists did not think of and publish a quantum chemical solution.^166−168^ These chemists included Jerome Berson, Breslow, William G. Dauben, Charles DePuy, Dewar, Doering, Roberts, and Zimmerman, all eventual members of the U.S. National Academy of Sciences, and several of whom^104,105,226,227^ had written books on quantum chemistry!

When that solution to the no-mechanism problem came in the only form it could, via quantum chemistry, it was regarded as a major discovery, worthy of the 1981 Nobel Prize in Chemistry to Hoffmann. Ironically, many physical chemists and theoretical chemists of that era, the 1960s, thought that the orbital symmetry solution was obvious—once you knew of the no-mechanim problem. And it was, if you knew quantum chemistry and organic chemistry and applied that typically bifurcated and isolated knowledge simultaneously. Quantum chemistry was simply not the “go-to” tool prior to the mid-1960s, at least not by organic chemists.

**Figure 5:**
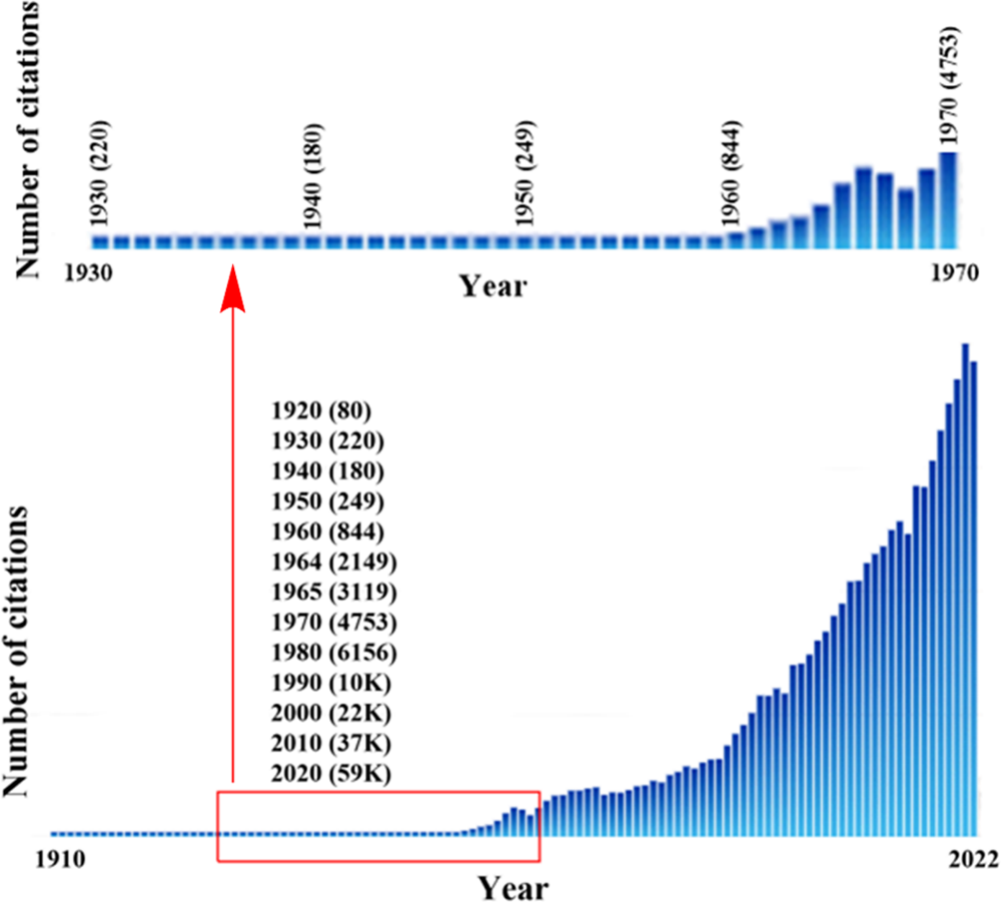
SciFinder^n^ “hits” for “quantum” from 1910 to 2022. “Hits’ refers to the number of publications in which the search term is found at least once in either the publication’s title, abstract, or concepts list in SciFinder^n^. The top graphic is a blow-up of the portion of the bottom graphic enclosed within the red rectangle.

Bibliographic analysis provides support for the impact of quantum chemistry on chemistry as a function of chronology from the 1930s through the 1960s.^228−232^ As seen in Figure 5, the appearance of the term “quantum” in the publication’s title, abstract, or concepts list of publications within the SciFinder^n^ database is low but not trivial, e.g., 220 instances in 1930 and 180 in 1940, until the counts rose, like an eruption, in the mid-1960s just when the Woodward–Hoffmann rules were published. Bibliographic analysis does not support a Quantum Chemical Revolution beginning in the late 1920s or early 1930s, nor does Figure 3. But bibliographic analysis does support an emerging revolution from the 1930s that erupted in the late 1960s.

Figure 5 suggests that the Woodward and Hoffmann rules were the precipitating agent for a dramatic paradigm shift in chemistry in the late-1960s. Please recall the discussion in section 2 regarding chronological time and cultural time.^41^ It is simply unreasonable to overlook the powerful chemical explanations provided by quantum chemistry to all sorts of experimental data prior to the Woodward–Hoffmann rules. The chronology indicated by Figure 3 and Figure 5 supports the proposition that the Quantum Chemical Revolution was a development that occurred over a 30-year period with the most dramatic step, the precipitating even, in terms of social impact on broad, community-wide chemical practice, being the Woodward–Hoffmann rules (Figure 1D). The term “social impact refers to three factors: (1) the arrival of computer technology, i.e., hardware and software, (2) the influx of vast amounts of experimental data due to the instrumental revolution (section 4), and (3) the enormous financial support of governmental support for university research.

In summary, the conversion of understanding, of deep understanding, of chemistry from a set of heuristic or “soft” explanations based on intuition and reasoning by analogy to explanations based on quantum chemistry^29^ is the essence of the Quantum Chemical Revolution. Achievements like Hückel’s rules were essential steps within that revolution. And the W–H rules were the tipping point of the Quantum Chemical Revolution.

## 9 Candidate 6: Retrosynthetic Analysis

**Figure 6:**
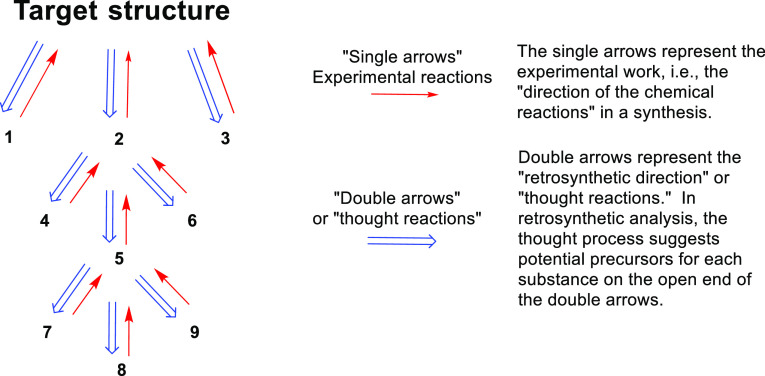
A generalization of E. J. Corey’s method of retrosynthetic analysis for synthesis illustrating a “tree” showing three first generation synthetic target structures (**1**–**3**), three second generation synthetic targets from **2** (**4**–**6**), and so on.^238,239,241,242,245−249^ Adapted and reproduced with permission from ref (84). Copyright 2018 John Wiley and Sons.

I now consider the sixth candidate for a revolution in chemistry, namely E. J. Corey’s method of planning organic syntheses: retrosynthetic analysis (Figure 6). Corey received the Nobel Prize in 1990 “for his development of the theory and methodology of organic synthesis.”^233−237^ In his Nobel Prize lecture,^238^ Corey characterized the methodology of organic synthesis prior to his development of retrosynthetic analysis as

“an automatic ‘know how’ rather than from the conscious application of well-defined procedures ... [and that the synthesis of complex compounds] seemed to be unique and to require a very high level of creativity and invention.”^238^

Corey surely was not suggesting that his achievement, retrosynthetic analysis, which has become the standard method of synthesis by experimental chemists ever since, including to this day, was to remove “a very high level of creativity and invention” from the required activities of chemists. Rather, what Corey had formulated was an organized methodology within which creativity and invention would be applied at a higher and more productive level of achievement.

**Figure 7:**
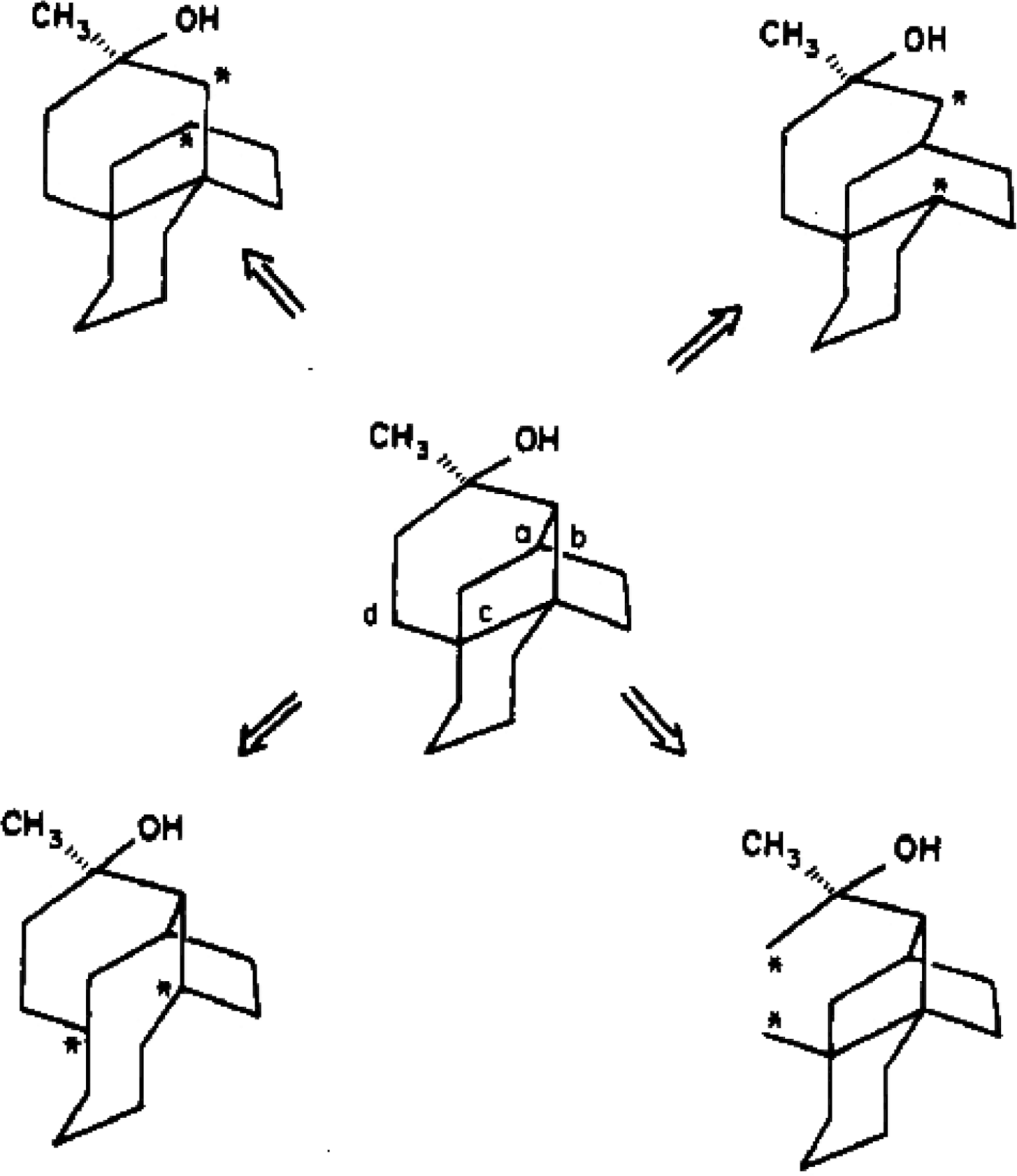
E. J. Corey’s first illustration of retrosynthetic analysis using double-headed arrows in 1975.^242^ This graphic appeared in his publication of LHASA-10, “the Harvard program for computer-assisted synthetic analysis, and it has been utilized in various ways for goal generation to guide antithetic operations.”^242^ Reproduced from ref (242). Copyright 1975 American Chemical Society.

Figure 6 illustrates Corey’s method of retrosynthetic analysis. The earliest of Corey’s working retrosynthetic analyses for his own research program was that devised for longifolene in 1957^239^ and achieved in 1961.^240,241^ This model was first described in retrosynthetic analysis terms with double-headed arrows and a protype “tree” in 1975 (Figure 7).^242^ Of course, decades before Corey (and James Hendrickson who also contributed to this field in the early 1970s^243^), Robert Robinson provided an early teaching of retrosynthetic planning in his biomimetic synthesis of tropinone in 1917.^244^

The challenges resulting from complex target structures demand from users of retrosynthetic analysis a “very high level of creativity and invention,” not less. Chemists need greater skill and increased determination as target structures have become more and more complex.^237,250−256^

Estimates of molecular complexity are now being developed in concert with retrosynthetic tools.^256,257^ After a lag several decades, Corey’s vision of computer-assisted synthesis via retrosynthetic analysis seems to be in the near future if not already here, especially benefiting from today’s powerful computer technology, the promise of artificial intelligence,^258^ and neural machine translations.^259^

**Figure 8:**
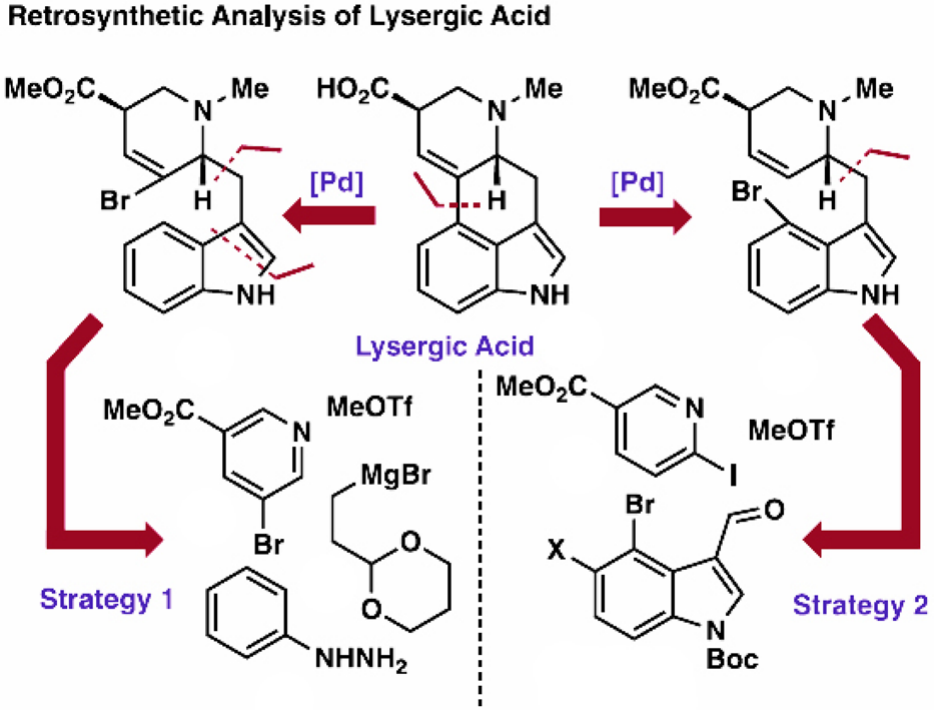
This graphic is a recent explicit example of the utilization of retrosynthetic analysis in a total synthesis, in this case, of lysergic acid.^260^ Reproduced from ref (260). Copyright 2023 American Chemical Society.

I believe that for many years in the past, today, and into the distant future, every chemist contemplating the synthesis of a complex target molecule used, uses, and will use some form of retrosynthetic analysis. For a very recent example, see Figure 8.

According to my evaluations shown in Table 1, as important as retrosynthetic analysis has been in bringing synthesis, not just total syntheses, into the hands of all chemists, retrosynthetic analysis did not initiate a revolution in chemistry (the last column in Table 1, four checks and three minor checks). Retrosynthetic analysis certainly changed the way most, if not all, chemists plan and perform synthetic chemistry. And there is the reasonable anticipation that retrosynthetic analysis will become vastly improved in the near future with AI, network analysis, and ever-enhancing software and computer power. Nonetheless, as indicated in Table 1, I do not judge the overall nature of chemistry to have been or to have the potential to become fundamentally changed as a result of retrosynthetic analysis. (But ultimately, artificial intelligence and data mining coupled with retrosynthetic analysis may become a component of a future revolution in chemistry.)

I return to Table 1 (column 7) and the lack of characteristics for a revolution in science for retrosynthetic analysis. There were no individual or community-wide disappointments with current theories in organic synthesis, and no state of crisis had occurred. No anomalies were resolved. There was no irreversible change in organizational structures in chemistry, and no new subdisciplines of chemistry arose. There was no taxonomic or lexiconic change with the exception that the term “retrosynthetic analysis” and its associated method were invented. Corey did propose^238,248,249^ the terms “carbogen”, “EXTGT”, “retron” and “partial retron”, and “transforms” to be used with retrosynthetic analysis. But while the tool itself is standard in chemistry, these terms have by and large vanished.

Table 1 shows seven characteristics that retrosynthetic analysis did have that relate to revolutions in chemistry. Retrosynthetic analysis was an important breakthrough in chemistry and continues to be a critical tool used by perhaps all synthetic chemists. The elaboration of retrosynthetic analysis was celebrated by the unshared Nobel Prize in Chemistry to Corey in 1990 “for his development of the theory and methodology of organic synthesis.” But, as noted above, a Nobel Prize does not, in and of itself, a revolution make.

I shall now comment on several of the characteristics in Table 1. Synthesis in organic chemistry was advancing in the 1950s and 1960s before Corey’s retrosynthetic analysis appeared. There were, of course, Woodward’s syntheses^261,262^ of quinine in 1944,^263^ patulin in 1950,^264^ cholesterol and cortisone in 1951,^265,266^ strychnine in 1954,^267^ lysergic acid in 1956,^268^ reserpine in 1956,^26888^ and chlorophyll in 1960^269^, among others; Karl Folkers’s synthesis of pyridoxine in 1939^270^ and pantothenic acid in 1940;^271^ Gilbert Stork’s synthesis of cedrene and cedrol in 1955;^272^ John C. Sheehan had synthesized penicillin in 1957;^273^ Eugene E. van Tamelen had synthesized yohimbine in 1958;^274^ van Tamelen^275^ and Albert Eschenmoser synthesized colchicine in 1959,^276^ and so forth. Three things these syntheses had in common: first, they were performed before Corey’s pronouncement of retrosynthetic analysis; second, their inventors uniformly failed to provide their synthetic planning, even at a basic level; and third, these chemists were among the elite of the profession.

I conclude that retrosynthetic analysis brought synthesis, both total synthesis of natural products and everyday synthetic challenges, to all chemists. It would no longer be an exploit only by the elite.

## 10 Discussion

### 10.1 What if You Do not Believe in Revolutions in Chemistry?

That is fine. But surely everyone believes in progress in chemistry.

Table 1 can be used as the springboard to evaluate and compare past and present events in terms of their their consequences. Table 1 is not intended to be the very last word on the characteristics of progress in chemistry but rather a starting place that is based on the hard work and deep thinking of many philosophers and historians of chemistry and chemists over a 60-year period. Do recall that Thomas Kuhn’s book *The Structure of Scientific Revolutions*(1) was first published in 1962, and I. B. Cohen’s book *Revolution in Science*(4) appeared in 1987. We have much to gain from learning from these and many other scholars about the progress in science.

Some may conclude that all progress in chemistry is evolutionary (Figure 1A) and does not incorporate disruptive changes and revolutions (Figure 1B–D). Certainly, some believe that definitions of human endeavors can cause barriers that inhibit thinking and imagination. I simply suggest that Table 1 can provide a useful tool to investigate progress in science and even plan and rank programs for funding. Used in its most flexible embodiment, Table 1 can distinguish between the immediate, medium term, and long-term impact of, for example, the Woodward–Hoffmann rules compared to Hückel’s rule.

One may equally well ask: was Boyle’s Law a revolution? Were Newton’s laws? Avogadro’s Law? The Bunsen-Kirchhoff Spectroscope? Einstein’s General Relativity? The Schrödinger Equation? One might say, “It is totally unimportant to know whether any or all of these were breakthroughs or revolutions. Rather, it is only important to know what they are about and, if one is interested in history, to understand their role in science and their place in relation to other discoveries.” I shall not argue with that position. But rather, I suggest using Table 1 in the evaluation of their impact. That individual may further argue, “I think each scientist is going to have her own ideas about what she considers important, paradigmatic, or revolutionary.” Again I suggest using Table 1 and discussing the individual characteristics of various achievements and their consequences. Surely the six candidates evaluated using Table 1 are not the same, in terms of their impact on science, on scientists, and on the social construction of science. Using the characteristics of Table 1, or even an enhanced version of Table 1, can provide a basis for discussion.

### 10.2 Are Characteristics of Revolutions in Chemistry from the Past Relevant to More Recent and Future Candidate Revolutions in Chemistry?

Can the same set of characteristics that captured the past revolutions (Table 1) be applied to present advancements and in the distant future? I provide two answers to this question.

Yes: The 13 characteristics found in Table 1 have come from research that evaluated revolutions in science from the 18th century to the present, i.e., over 300 years of candidate revolutions in science. Since so much has changed in science in the past 300 years, one might anticipate that at least the major characteristics of revolutions in chemistry will have been identified. These are contained in Table 1. And in this study, I have used these characteristics to interrogate six candidate revolutions in 20th century chemistry.

No, or at least not completely: There are innumerable examples in which researchers try to extrapolate beyond their data set. Doing so into the far chronological future is, as we all know, extremely risky at best. I do specify that users of Table 1 must be prepared to be flexible, to add and to remove or downgrade characteristics that are not useful in some future era. It is as simple as that. But in a comparison of revolutions over more years than we have today, it might be wise to retain criteria in Table 1 that have been shown to be useful for revolutions of the past.

### 10.3 One Revolution in Chemistry Synergized a Second Revolution

A revolution in chemistry can certainly involve one or more major breakthroughs in chemistry. I also believe that a revolution in chemistry can stimulate, even participate in a second revolution in chemistry. For example, the 20th century Instrumental Revolution provided an enormous database of valence isomerizations, i.e., reactant-product stereochemical relationships, seen and unseen, i.e., allowed and forbidden, reactions, that led to the Woodward–Hoffmann rules and ultimately the dramatic appearance of the Quantum Chemistry Revolution.

### 10.4 Can There Be Too Many (or Too Few) Revolutions in Chemistry?

Some might argue that the number of revolutions is small, and that it should be small, because, if there were too many revolutions, they would lose their significance. But while the number of days and years in a century stay constant, the rate of progress in chemistry during the past several centuries surely has increased dramatically. Surely chemical research is performed much more efficiently today, e.g., the structure of strychnine was determined by tens of chemists during a 40-year period in the first half of the 20th century;^80^ today, an X-ray analysis might be achieved within 24 h. Furthermore, there are far more chemists performing far more research in far more institutions funded by far more physical and financial resources in far more countries than ever before. Furthermore, the discipline of chemistry has grown to include much more than “pure chemistry.” I need not list the long tentacles of chemistry, the central science.

I conclude: one revolution in chemistry in the 18th century is reasonable. I further conclude: because of the exponential growth of chemistry in the 20th century, two or even more revolutions in chemistry, the molecular sciences, and chemistry-related life sciences are reasonable in the 21st century. (I include “chemistry-related life sciences here, just as the Nobel Foundation has done^232^ so, as have many one-time Departments of Chemistry have become the Department of Chemistry and Chemical Biology (Harvard University) and the Department of Chemistry & Biochemistry (UCLA).

### 10.5 Predictions of Future Revolutions in Chemistry

Are we in the midst of any revolutions in chemistry today? Chemists involved in revolutions may well not recognize they’re in it until one is well underway. Why? Because one is so involved in the science, there may be no time or even the incentive to stand outside of one’s own current experiences and judge, looking inward.

Where are the major opportunities for a revolution in chemistry today, based on the characteristics in Table 1? Hints come from the recent Nobel Prizes. Perhaps genome editing technology using CRISPR or some other future technology to do *in vivo* chemistry. Perhaps a revolution in terms of data mining and the utilization of enormously large databases and tools such as artificial intelligence and neural networks. And then there are societal drivers, including urgent needs for breakthroughs dealing with energy, global warming, new pharmaceuticals, dwindling resources especially of water, rare earth elements, and even lithium for batteries, drought- and pest-resistant crops, green chemistry, among many others. Others have their lists, too.^277−279^ The examples are of different kinds. Some of these are in terms of what people study; others are in terms of how science is done.

One final thought on predicting future revolutions in chemistry. We do such predictions all the time, though mostly by intuition, not using some formalized set of criteria as embodied in Table 1. We attempt to predict the future when we select individuals for entrance to colleges and universities (at every level); when we decide who gets tenure or not and which grant proposals receive funding; and when we choose articles for inclusion in our journals, deciding on their value for future readers and the archives of science. The Fields Medal was originally designed to choose the most promising young mathematicians, not those who were already anointed as stars.^280^ In 1997, *Pure and Applied Chemistry,* IUPAC’s flagship journal, ran an entire issue on the topic “Physical Organic Chemistry for the 21st century.”^281^ Such elite physical organic chemists as Breslow,^282^ Marye Anne Fox,^283^ George Hammond,^284^ Kendall N. Houk,^285^ Keith Ingold,^286^ Roberts,^287^ Streitwieser,^288^ and Frank Westheimer^289^ engaged with this topic and provided their predictions for the future of the field. I posit that using the criteria in Table 1 can assist in guiding one’s predictions of the future of chemistry and perhaps even our own research agenda and professional strategic plans.

### 10.6 Future Applications and Modifications of Table 1

Does the definition and process framework (Table 1) apply in scientific fields other than chemistry? I think so, in large measure because I obtained the characteristics listed in Table 1 from an assembly of publications that treated several different scientific disciplines. Table 1 is intended to be a working construct, just as provided in its first applications.^22^ The content of Table 1 and the manner in which it can be used is not prescriptive. However one modifies and uses Table 1, the process remains qualitative, descriptive, and flexible. While resultant judgements will always be arbitrary and sensitive to biases, I claim that all together, Table 1 provides a reasonable basis for comparing a set of candidate revolutions. Errors tend to compensate for each other, leading to reasonable judgments.^290^ This belief is especially valid when comparing several candidate revolutions with each other.

## 11 Conclusions

### 11.1 Revolutions in Chemistry Do Occur

Table 1 reveals that progress in chemistry can be delineated by assessing a series of characteristics that describe revolutions in science. There are certainly periods in time in which assessment of the characteristics listed in Table 1 describe normal, evolutionary progress in chemistry (Figure 1A). And there are much rarer episodes in chemistry for which such assessments describe revolutions in chemistry (Figure 1B–D). Revolutions incorporate a discontinuity and rupture in chemical knowledge coupled with significant consequences within the social construction of chemistry. The identification of both normal, evolutionary progress as well as revolutionary growth is sufficient evidence for me to conclude that there are, indeed, such growth bifurcations in chemistry. I now summarize several major conclusions from this research.

### 11.2 On the Identification of Revolutions in 20th Century Chemistry

In this publication, I have evaluated six 20th century candidate revolutions in chemistry using Table 1. My analysis supports the conclusion that there was an Instrumental Revolution in chemistry in the 20th century. The assignment of the “Instrumental Revolution” as a revolution in chemistry is due to its four previously undiscussed properties: (i) In the 20th century, there was mindful innovation and production of many instruments, each specifically designed to solve a specific, high-quality and pressing research problem. (ii) The overwhelming amount of analytical data provided by these instruments vastly increased the knowledge and understanding of all subdisciplines of chemistry. (iii) The intellectual and social consequences of this new knowledge were immense. (iv) The new instruments provided new types of data that led to new concepts, new projects, new questions, and thus an avalanche of even more data. The subdiscipline of analytical chemistry was born along with new journals and new organization structures, especially within the chemical industry. I also conclude that there was no particular precipitating event or “tipping point instrument” that caused an eruptive revolution in science. The Instrumental Revolution is likely best represented by Figure 1B.

I also posit that there was a Quantum Chemistry Revolution that began in the late 1920s or early 1930s and extended into the mid-1960s. Quantum chemical explanations were for a variety of physical properties including states of matter as well as for chemical bonding and chemical reactivity. But there’s a caveat: To a large extent and especially for organic chemists, quantum chemistry was a sleeping beauty in chemistry.^121,122^ Two pieces of evidence support the sleeping beauty description of quantum chemistry. First, there were very few publications having abstracts that included either “quantum” or “molecular orbital” or “mol. orbital” until the mid-1960s. Second, quantum chemistry was hardly used to explain chemical reactivity until the mid-1960s when the Woodward–Hoffmann rules were formulated to explain the mechanism of cyclic concerted reactions.

The Quantum Chemical Revolution in the guise of the W–H rules in the mid-1960s demonstrated the synergy between theoretical chemistry/computational chemistry and experimental chemistry. A new subdiscipline of chemistry was born, that being computational chemistry. New journals specializing in computational chemistry were founded, and even the conservative journals such as *JACS* began to publish articles that were entirely computational chemistry, i.e., that had no new experimental results. New organizational structures were born, especially in the pharmaceutical industry, centered on computational (medicinal) chemistry. The Quantum Chemical Revolution is likely best represented by Figure 1D.

Inspection of Table 1 reveals that the two revolutions in 20th century chemistry as discussed above do incorporate the same characteristics. However, analysis of many of their shared characteristics indicates significant fine differences between the two revolutions. This is not unexpected. Focused studies on specific revolutions in science can and have provided distinguishing characteristics unique to each.

As described above, not all revolutions in chemistry, in science, require a precipitating event or a tipping point discovery. But I also tentatively conclude that for those that do embody a precipitating event, that precipitating event may well have the identical check marks in Table 1 as does the revolution itself. Further research is required to establish if this as a valid generalization.

### 11.3 What is the Difference Between a “Precipitator” of a Revolution in Science versus a “Revolution in Science”? A Second Unanticipated Value

In section 4.4, I discussed an unanticipated value of analyses of revolutions in science and candidates for revolutions in science. I now make another admission, a not unwelcome feature for a Perspective in *JACS Au*.

During my research on the history of the Woodward–Hoffmann rules and especially during my writing a series publications on that topic,^291−293^ I came to perceive that the W–H rules had irreversibly transformed chemistry. I imagined that the W–H rules constituted, like the Instrumental Revolution, a revolution in chemistry. This vision led me in two directions. First, I had to immerse myself in the rather vast literature of revolutions in science. I quickly discovered that, in spite of many books and publications on the subject, there was no uniformly agreed upon definition of “revolution in science.” That understanding ultimately led to Table 1 which was first published in a philosophy of chemistry journal.^22^ Second, I needed to study, and did so with Dean Tantillo who joined in that adventure, the bifurcation of explanations in chemistry between “soft” and “hard” theories, i.e., between heuristic, intuitive explanations and explanations based on quantum chemistry.^29^ With that knowledge in hand, I applied Table 1 to the Woodward–Hoffmann experience. I assigned 13 checks for W–H in Table 1 and thus concluded that there was a Woodward–Hoffmann Revolution in chemistry.

But readers of early and more advanced versions of this publication pushed back, and they did so with vigor. They claimed that W–H was not so important; it was quantum chemistry. Having used bibliographic analyses for other projects dealing with the progress of chemistry,^232,294,295^ I rushed to obtain information in part contained in Figures 3 and 5. My immediate conclusion from those two figures was to reject the assertions of my colleagues. But I also knew that they were right. I knew that spectroscopists among others had relied on quantum chemistry, that “soft” theories were horribly insufficient to explain an increasing number of experimental results. But when I looked closer at the bibliographic data, there indeed were “hits” prior to Woodward and Hoffmann. Of course, there had to be!

So, in terms of revolutions in chemistry, what was the relationship between the W–H rules and quantum chemistry? They both led to 13 checks in Table 1. And then the answer was evident, just as a hindsight critic asserts that the solution to the no-mechanism problem was obvious, immediately after reading the W–H publications. Once you know the answers, the solutions to many problems often do seem obvious. After additional analyses and some deep thinking, I came to the conclusion that my colleagues’ insights were on the mark. I then concluded that the W–H rules were the tipping point, the precipitating chemistry that brought the Quantum Chemistry Revolution into full steam, irreversibly and with full life and spirit. On this basis, I conclude that the Quantum Chemistry Revolution looks like Figure 1D.

### 11.4 Are There “Must Have” Characteristics of Revolutions in Chemistry? The Role of Social Construction of Chemistry

A comparison of these six candidates has revealed that several factors seem to be required for a revolution in chemistry. These are as follows: (i) paradigm shifts in either concept or in the practice of chemistry are necessary for a revolution in chemistry, (ii) a consequential intellectual component associated with that paradigm shift, and (iii) a major social construction of science component, to its initiation and/or for its consequences.

I believe that all revolutions in chemistry, and perhaps all revolutions in science in general, must have a significant involvement in the social construction of knowledge (factor 5 in Table 1). I believe that a seminal breakthrough in science *alone* will never result in a revolution in science unless it also contains a significant effect on the context in which chemistry is practiced. This may be in terms of the organization of the practice of science, its methodology and equipment (instrumentation, computer capabilities, and other resources), its connectivity with the nonscientific community, its effect on science education, the creation of new journals and awards, and so forth.

### 11.5 What is the Difference Between Breakthroughs in Chemistry and Revolutions in Chemistry?

It is interesting to consider that there is potentially a difference between discoveries and inventions being transformative in a science and being revolutionary. A revolutionary discovery is not necessarily sufficient to initiate a revolution in science.

The major though not only distinction between a breakthrough achievement in chemistry, for which there is even an award, the Citation for Chemical Breakthrough Award (CCB Award) presented by the Division of History of Chemistry (HIST) of the American Chemical Society,^296^ and a revolution in chemistry is the factor dealing with the social construction of knowledge. Table 1 lists other characteristics of revolutions in chemistry that are typically not met by even the most compelling scientific breakthroughs in chemistry but are seen in scientific revolutions. As discussed above, Hückel’s rule and Corey’s retrosynthetic analysis were breakthrough achievements in chemistry, i.e., point achievements, but neither is judged by this author as initiating a revolution in chemistry. In both of these examples, there were no major social initiating causes or social consequences among other lacking characteristics of revolutions in science which led me to conclude: breakthroughs, most certainly yes; revolutions in chemistry, no.

### 11.6 On the Method for Assessing Revolutions in Chemistry

For many decades, chemists, historians, and philosophers of science have discussed revolutions in chemistry.^1,2,22,297−299^ But they have done so in the absence of any formulated, consistent, and portable definition of this concept. In a recent publication, I identified hundreds of characteristics of revolutions in science that have appeared in the literature,^22^ many of which were close relatives to each other. That analysis led to a working and ever evolving definition of revolutions in chemistry, currently consisting of 13 independent characteristics that were grouped into six independent factors (Table 1). This research has also demonstrated the value of performing parallel evaluations of several candidates as well as comparing several candidate revolutions in science with each other. Such a multiple, simultaneous evaluation can (i) highlight the strengths and weaknesses of each candidate relative to the characteristics in Table 1 and (ii) provide support or otherwise regarding the addition or exclusion of various characteristics in Table 1.

### 11.7 On the Use of Characteristics of Revolutions in Chemistry for Other Assessments Including the Strategic Planning of One’s Own Research Trajectory

One of the strengths of Table 1 is using its characteristics and factors in evaluating candidate revolutions. Other uses of Table 1 include the analysis of ongoing events in chemistry, in the identification of current revolutions in chemistry and the prediction of future revolutions in chemistry, and in the analysis and planning of individual research trajectories. The optimal usage of Table 1 is in an intense and multiple-pass iterative inquiry in the evaluation of each of its included characteristics.

Just as revolutions incorporate discontinuity and rupture in chemical knowledge and practice, historical analysis of many individual research programs reveals discontinuity and rupture in research goals and methodologies and location. This is most evident in the careers of certain eminent chemists. Derek H. R. Barton commented that he changed research programs whenever he was in command of the entire literature of that area of chemistry.^300−302^ Barton also moved to different universities that were located in quite different cultural settings: from England to Scotland and back to England, to France, and then to Texas. Teruaki Mukaiyama believed it necessary to change research directions every five years.^303,304^ Some chemists transferred from industry to academia or the converse (or, like Robert E. Ireland, James P. Snyder, and Fred Wudl, from academia to industry and then back to academia).

### 11.8 On the Relevance of the History, Sociology, and Philosophy of Science in Scientific Research

Try it yourself, you will see its relevance. Indeed, your reading this publication is a simple test. Has it provided you with any insights or questions or even energy and excitement?

I have argued^37^ that journals like *Chemical Reviews*, *Accounts of Chemical Research*, and *Chemical Society Reviews* and books like *Organic Reactions* are history of chemistry. So is every introduction to every chemistry journal publication that reviews the literature. It is impossible to imagine presenting new research findings in the absence of context, in the absence of a pertinent literature review. With that perspective, we all are historians of our own science.^30500^

In 2019, Lucie Laplane, Hasok Chang, and others published an essay in the *Proceedings of the National Academy of Sciences* entitled “Why Science Needs Philosophy.”^306^ These authors offered several suggestions as to the actions that scientists and philosophers could do in order to benefit their own research and, more generally, science and the history and philosophy of science. A recent book by the theoretical astrophysicist and Nobel laureate (2019 in physics) P. James E. Peebles discusses the value of the sociology and philosophy of science in scientific research.^307^ Underlying the history, philosophy, and sociology of science is the science itself. They are all discussing and teaching the same phenomenon though from different perspectives.^37,305,308^ In a real sense, the study of one is a study of all.^309^

Two reviewers of this manuscript have asked, “It would be relevant to hear more about how this analysis might impact current and future training and research in chemistry.” It is trite to say that including history and philosophy of science in today’s undergraduate and graduate chemistry courses is a topic much discussed, especially by historians and philosophers of science hoping to influence practicing educators. My own colleagues at the University of Richmond, a PUI (primarily undergraduate institution), have on occasion asked me to present a lecture on a designated topic in the history of chemistry that was relevant to their special course syllabus. But calls for the inclusion of history of science in their curricula, few universities these days have historians of science who can insert themselves seamlessly into a current science course.

While I have not performed research on this aspect of pedagogy, I can offer what I have heard over many years, including from Brian Coppola, a scholar for many years active in improving pedagogy in chemistry.^310^ “Too much to teach, not enough time.” “Insufficient resources.” “I don’t have that expertise beyond an anecdotal tale or two.” “I am uncertain as to how to integrate history of chemistry into instruction so as to improve understanding.” “Few students will sign up for a course taught by a historian of science.” “Students are focused primarily on grades, not on learning.” I will add that several recent textbooks in organic chemistry have included history of chemistry as a minor yet noticeable feature in their text.^311,312^

## 12 What if You Disagree with My Conclusions? That’s Fine (As Long As You Follow Table 1 or Your Own Modification of Table 1)

Please do not throw out the baby with the bath water. There is a difference between consequential, seminal breakthroughs in chemistry, of which there are not many,^296^ and revolutions in chemistry, of which there have been scarcely any (section 11.3). You may disagree with my judgments of candidate revolutions. Fine. I admit to the subjectivity of my decisions; the reader might devise alternatives. You are encouraged to choose your own candidates for evaluation. The strategy is to apply Table 1 or a modification thereof to candidate revolutions and to learn from such applications, not to discard Table 1 based solely on my choices and judgments reflected in this publication.

You may disagree with some of the characteristics listed in Table 1. You may disagree with my specific ratings regarding any or all of the characteristics listed in Table 1 for certain of the candidates. You may disagree with my evaluations as to which of the six candidates examined herein were revolutions in chemistry. Fine. Then identify additional characteristics that you consider to be mandatory or sympathetic for a revolution in chemistry and add them to Table 1. But keep in mind: the characteristics in Table 1 are not mine. They have longevity and legs. They have portability, as they were exported from the vast literature on revolutions in science.

Judgments and classifications of revolutions in chemistry are subjective, mine included. But such judgments should be based on carefully chosen, itemized, and well-defended characteristics, not on wandering banter and effusive text. I suggest the use of Table 1 as it incorporates the characteristics provided by numerous scholars in their studies on revolutions in chemistry over the past 60 years. Arbitrary and unreflective elimination of characteristics from Table 1 should be avoided. Optimally, several candidate revolutions should be discussed and simultaneously evaluated, as this defuses specific biases and minimizes arbitrary arguments.

I hope Table 1 will be called upon to contribute to the history and philosophy of chemistry in the future. I predict an interesting and perhaps even instructive exercises will result.

## 13 Coda: On Metaphors and Revolutions in Science

Upon reading an advanced draft of this publication, Roald Hoffmann commented,

“I would note in passing that the terms you effectively use in your analysis, namely

A. Revolution;

B. Tipping point;

C. Precipitating factor; and

D. Irreversible

are three nouns and an adjective, motion implicit in all. The terms are pulled into service by you and, of course, by others in an analysis of the history and philosophy of science. Their origins are diverse: A, from history and the description of social change; B, from physics; C, from chemistry; and D, from physics and chemistry. Yes, in the process of being used as metaphors, these terms lose some of the originating meanings or connotations, and they acquire new ones.

But it may be good to keep in mind that perhaps they don’t lose as many associations (to their origins) as one might imagine.

Note also that deep philosophical questions underlie the application of these four metaphors, questions that human beings have pondered for millenia. I see four: 1. Space and reality, where things are located; 2. Their motion: its time, rate of change—both in the sense of velocity and acceleration; 3. Continuity or discontinuity of the process; and 4. The very chemical notion of equilibrium and of perturbing it.

These philosophical questions are important, fascinating. But chemistry did not and does not wait for them to be resolved.”^313^

## Dedication

Dedicated to the memory of Joseph F. Bunnett (1921–2015), an eminent physical organic chemist and the founding and longtime editor-in-chief of *Accounts of Chemical Research.* Bunnett provided the catalysis that began my own professional revolution into the history of chemistry.
